# Syncope after aortic valve surgery

**DOI:** 10.1007/s12471-019-1242-5

**Published:** 2019-02-11

**Authors:** C. Crooijmans, L. M. Rademakers

**Affiliations:** Department of Cardiology, Catharina Ziekenhuis, Eindhoven, The Netherlands

A 49-year-old man with a bicuspid aortic valve with severe stenosis and moderately depressed left ventricular ejection fraction underwent implantation of a mechanical aortic prosthesis. During the pre-operative evaluation, the electrocardiogram (ECG) showed sinus rhythm with first-degree atrioventricular block and voltage criteria of left ventricular hypertrophy (Fig. 1).

**Fig. 1:**
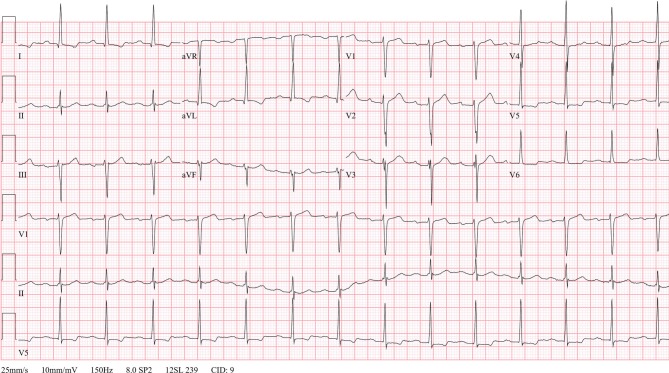
ECG during pre-operative evaluation

On the fourth postoperative day, the patient complained of sudden onset of rapid palpitations. The ECG is shown in Fig. 2. Both carotid sinus massage and a rapid intravenous bolus of 20 mg adenosine did neither temporarily slow down nor stop the tachycardia. Meanwhile, his blood pressure had declined to 70/40 mm Hg. A few minutes later, the patient lost consciousness and underwent immediate direct-current (DC) cardioversion. What is your diagnosis?

**Fig. 2:**
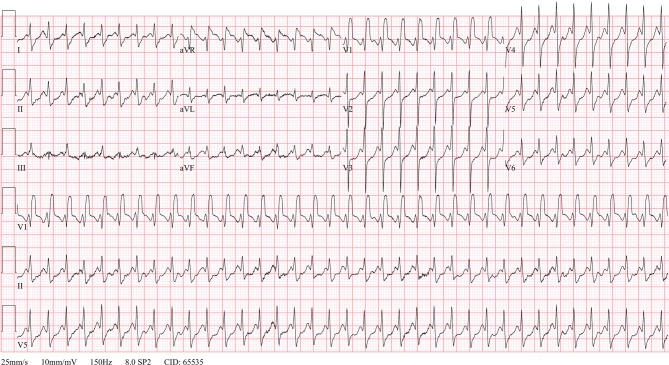
ECG during rapid palpitations

## Answer

You will find the answer elsewhere in this issue.

